# Clinical impact of molecular breast imaging as adjunct diagnostic modality in evaluation of indeterminate breast abnormalities and unresolved diagnostic concerns

**DOI:** 10.1097/MNM.0000000000001684

**Published:** 2023-03-10

**Authors:** Ariane A. van Loevezijn, Christinne L.S. Corion, Anneke M. Zeillemaker, Lidy M.H. Wijers, Robin H.M. Smithuis, Renato A. Valdés Olmos, Jos A. van der Hage, Lioe-Fee de Geus-Oei, Menno Benard, Lenka M. Pereira Arias-Bouda

**Affiliations:** a Department of Surgical Oncology, Netherlands Cancer Institute – Antoni van Leeuwenhoek, Amsterdam; b Department of Surgery, Haaglanden Medisch Centrum, Den Haag; c Department of Surgery; d Department of Radiology, Alrijne Hospital, Leiderdorp; e Department of Radiology, Section of Nuclear Medicine; f Department of Surgery, Leiden University Medical Centre, Leiden; g Biomedical Photonic Imaging Group, University of Twente, Enschede; h Department of Research and Education, Alrijne Hospital; i Department of Nuclear Medicine, Alrijne hospital, Leiderdorp, The Netherlands

**Keywords:** breast-specific gamma imaging, breast cancer, diagnostic imaging, molecular breast imaging, ^99m^Tc-sestamibi

## Abstract

Improvements in molecular breast imaging (MBI) have increased the use of MBI as adjunct diagnostic modality and alternative to MRI. We aimed to assess the value of MBI in patients with equivocal breast lesions on conventional imaging, especially in terms of its ability to rule out malignancy.

**Methods:**

We selected patients who underwent MBI in addition to conventional diagnostics due to equivocal breast lesions between 2012 and 2015. All patients underwent digital mammography, target ultrasound and MBI. MBI was performed using a single-head Dilon 6800 gamma camera after administration of 600 MBq ^99m^Tc-sestamibi. Imaging was reported according to BI-RADS classification and compared with pathology or follow-up of ≥6 months.

**Results:**

Of 226 women included, pathology was obtained in 106 (47%) and (pre)malignant lesions were found in 25 (11%). Median follow-up was 5.4 years (IQR 3.9–7.1). Sensitivity was higher for MBI compared to conventional diagnostics (84% vs. 32%; *P* = 0.002), identifying malignancy in 21 and 6 patients, respectively, but specificity did not differ (86% vs. 81%; *P* = 0.161). Positive and negative predictive value were 43% and 98% for MBI and 17% and 91% for conventional diagnostics. MBI was discordant with conventional diagnostics in 68 (30%) patients and correctly changed diagnosis in 46 (20%) patients, identifying 15 malignant lesions. In subgroups with nipple discharge (*N* = 42) and BI-RADS 3 lesions (*N* = 113) MBI detected 7 of 8 occult malignancies.

**Conclusion:**

MBI correctly adjusted treatment in 20% of patients with diagnostic concerns after conventional work-up, and could rule out malignancy with a high negative predictive value of 98%.

## Introduction

Mammography is the current gold standard in breast cancer screening, and evaluation of mammography-detected breast abnormalities is primarily performed with target ultrasound. The accuracy of mammography and target ultrasound; however, strongly depends on several factors such as breast density, the presence of breast implants or a history of breast surgery or radiotherapy [1,2]. Women with dense breast tissue or other factors that complicate evaluation with mammography may therefore benefit from supplemental imaging, such as MRI [3]. Dynamic contrast-enhanced MRI is highly sensitive for the detection of breast cancer and additional foci, but overall specificity is moderate [4–7]. Therefore, extra diagnostic procedures are regularly needed when undefined MRI lesions are found [6–9].

Recently, molecular breast imaging (MBI) with ^99m^Tc-sestamibi as a radiotracer has regained attention as supplemental imaging procedure. The uptake of ^99m^Tc-sestamibi in breast tumours is based on neoangiogenesis and increased mitochondrial density, which is captured by a gamma camera. In contrast to anatomical imaging, MBI is not limited by structural distortions or breast densities. Breast-dedicated gamma cameras nowadays are able to detect small-breast lesions (≤1 cm) at a relatively low-radiation dose, not affected by breast density [10–14]. Promising data have demonstrated that MBI is as sensitive as MRI (89%–97%) but show higher specificity rates (77%–89%) [12,15–19]. MBI is therefore increasingly being proposed as a useful alternative to MRI. Few studies; however, have reported on the clinical utility of MBI as supplemental imaging technique.

The aim of this study was to determine the diagnostic accuracy of MBI as a problem-solving modality in patients with equivocal or inconclusive breast abnormalities at conventional diagnostic work-up and to assess the impact of MBI on the subsequent treatment strategy. In addition, we evaluated patient characteristics associated with breast cancer and assessed the value of MBI in subgroups of patients with nipple discharge and BI-RADS 3 lesions.

## Material and methods

### Patient population

We included all women of ≥18 years who underwent MBI because of undefined breast lesions or equivocal findings at clinical examination and conventional diagnostic imaging between March 2012 and February 2015. The average period interval between conventional diagnostic and MBI was 2 weeks. Patient data were retrospectively collected and assessed. Exclusion criteria were histopathological confirmed (pre)malignant breast lesion prior to MBI and incomplete medical files. This study was approved by the local regulatory authorities.

### Imaging techniques

Conventional diagnostic imaging of the breasts consisted of a standard digital craniocaudal and medio-latero-oblique mammographic view (Siemens Inspiration Mammomat, Munich, Germany), followed by target ultrasound (Philips Affiniti 70 G Linear transducer L 12-5, Eindhoven, the Netherlands). Additional imaging with contrast-enhanced MRI (1.5-T system; Philips Ingenia, Eindhoven, the Netherlands) was only acquired if considered necessary at multidisciplinary consultation. Mammography, target ultrasound and MRI were interpreted by radiologists according to the American College of Radiology Breast Imaging Reporting and Data System (BI-RADS) Atlas [20]. Findings were categorised as normal (BI-RADS 1), benign, (BI-RADS 2), probably benign (BI-RADS 3), suspicious (BI-RADS 4), or highly suggestive of malignancy (BI-RADS 5).

MBI was conducted using the single-head Dilon 6800 gamma camera (Dilon Technologies, Newport News, Virginia, USA). Patients received an intravenous injection of 600 MBq ^99m^Tc-sestamibi 5–10 min prior to the imaging in a contralateral antecubital vein. Subsequent images were acquired in the craniocaudal and latero-oblique direction, comparable to the mammographic projections. If relevant, additional planar images (lateromedial or mediolateral view or axillary craniocaudal view) were acquired from the ipsilateral breast. The functional images were directly compared with the most recent mammography. Images were assessed using the image interpretation criteria as defined by the MBI Lexicon [21], referring to background activity, mass or non-mass uptake, intensity, internal pattern of uptake, location, distribution, and symmetry. Final assessment, based on these criteria, was made according to assessment codes modelled after those used in BI-RADS for other breast imaging modalities, tailored to MBI.

Histopathological analysis by fine-needle aspiration cytology (FNAC) or biopsy (ultrasound-guided, stereotactic, MRI-guided, or MBI-guided) of detected breast lesions was obtained in case of suspect or equivocal lesions whenever possible. MRI- or MBI-guided biopsy was performed only if the breast lesion could not be identified with target ultrasound. In addition to histopathological evaluation, representativeness of the biopsy specimens was assessed by measuring the activity in the tissue samples ex-vivo, directly after biopsy, using the parallel-hole collimator [22]. Follow-up imaging was conducted on a time interval considered clinically necessary. For analysing purposes, ductal carcinoma *in situ* was considered a malignant lesion and at least 6 months follow-up imaging had to be available in patients without a final pathological diagnosis.

### Outcomes

The primary outcome was the diagnostic accuracy (i.e. sensitivity, specificity, negative predictive value, and positive predictive value) of MBI, which was compared to the diagnostic accuracy of conventional imaging with mammography and the target ultrasound. For analysing purposes, BI-RADS categories 1–3 were considered negative for malignancy and categories 4–5 were considered positive for malignancy. Diagnostic imaging was classified as true-negative, false-negative, true-positive and false-positive for malignancy based on pathological findings or follow-up for at least 6 months.

Second, the clinical impact of MBI as adjunct modality after conventional work-up was assessed as the proportion of patients in which MBI led to correct upstaging or downstaging (i.e. correct change in BI-RADS > 3 or BI-RADS ≤ 3 classifications). In addition, patient characteristics associated with malignancy were assessed and subgroup analyses were performed for patients with nipple discharge and BI-RADS 3 lesions.

### Statistical analysis

The BI-RADS classification at conventional work-up and the BI-RADS classification of MBI were compared within patients using the Wilcoxon signed-rank test. The two-sided 95% confidence intervals for proportions were calculated using the Clopper–Pearson exact method. Differences in sensitivity and specificity between conventional imaging and MBI were analysed using McNemar’s test. Logistic regression was used to evaluate associations between baseline patient characteristics and breast malignancy. Subgroup analyses were prespecified for BI-RADS 3 lesions and patients with nipple discharge. Statistical significance for comparisons between groups was defined as *P* < 0.05. Statistical analysis was carried out using IBM statistics SPSS, version 25, Armonk, Ney York.

## Results

### Patient population

MBI was performed in 275 patients because of diagnostic concerns. Of these, 34 patients did not meet inclusion criteria, 4 patients had missing data, and 11 patients could not be analysed due to the absence of both histopathological confirmation and follow-up. In total, 226 patients were included for analysis. Median age was 54 years (range 21–86). Ninety (40%) patients were referred by the national breast cancer screening program and 136 (60%) patients presented with clinical symptoms. Baseline patient characteristics and indications for MBI are presented in Table 1.

**Table 1:** Baseline patient characteristics (n = 226)

Age (years)	54	(45–67)
History of breast cancer	40	(18%)
Positive family history	53	(24%)
Referral by breast cancer screening program	90	(40%)
Hormonal status		
Premenopausal	63	(28%)
Perimenopausal	26	(11%)
Postmenopausal	131	(58%)
Unknown	6	(3%)
Symptoms		
No symptoms	99	(44%)
Pain	14	(6%)
Palpable mass	60	(26%)
Skin abnormality	11	(5%)
Nipple discharge	42	(19%)
Clinical classification		
No abnormalities	114	(50%)
Benign	55	(24%)
Uncertain benign	36	(16%)
Uncertain malignant	21	(9%)
Breast density		
a.Mostly fatty	37	(16%)
b.Some density (scattered)	92	(41%)
c.Moderately dense (heterogeneous)	81	(36%)
d.Extremely dense	16	(7%)
Indication MBI		
BI-RADS 3 lesion	49	(22%)
Discrepancy with clinical findings	44	(19%)
History of breast surgery and equivocal lesion	24	(11%)
Mammography-detected lesion, occult with ultrasound	63	(28%)
MRI-detected lesion, occult with ultrasound	7	(3%)
Nipple discharge and equivocal or negative mammography + ultrasound	39	(17%)

Data are median (IQR) or *N* (%).

All patients underwent mammography and target ultrasound prior to MBI. Median BIRADS classification at conventional diagnostic imaging was 3 [interquartile range (IQR) 2–3] and median BIRADS classification with MBI was 2 (IQR 1–3) (*P* = 0.001).

Fourteen patients (6%) received additional imaging with both MBI and MRI. Four patients received additional imaging with MRI prior to MBI; MBI was performed additionally due to remaining clinical concerns. In one of these patients, MRI was technically unsuccessful (due to claustrophobia). The other three patients had lesions that were considered benign on MRI. In these three patients MRI- and MBI-findings were concordant. In two of three patients, the benign nature was confirmed by histopathological analysis. The third patient remained in follow-up without evidence of malignancy during follow-up.

Ten patients underwent MRI after MBI (due to remaining clinical concerns). In one patient a lesion was considered malignant by both MRI and MBI; histopathology; however, revealed benign aetiology. One patient had FNAC prior to MBI/MRI, showing malignant cells. Both MBI and MRI did not show a suspicious lesion; however, and both classified the lesion as benign. This patient remained in follow-up, without evidence of malignancy. In the remaining eight patients MRI-findings and MBI-findings were concordant as well: both modalities classified all lesions as benign and benign nature was confirmed by histopathological analysis in six of eight patients. Two patients remained in follow-up without evidence of malignancy during follow-up.

### Histopathological analysis

Histopathology was obtained in 106 (47%) patients. Of these, 47 (21%) patients underwent FNAC only, 23 (10%) patients had ultrasound-guided core biopsy with or without FNAC, 17 (8%) patients underwent MBI-guided biopsy, 14 (6%) patients had stereotactic vacuum-assisted biopsy, 2 (1%) patients had MRI-guided biopsy, and 3 (1%) patients underwent diagnostic excision.

At histopathological evaluation, a total of 25 (11%) patients were diagnosed with malignant breast lesions. Fifteen patients had invasive ductal carcinoma, four patients had invasive lobular carcinoma, five patients had ductal carcinoma *in situ* (DCIS), and one patient was diagnosed with intracystic carcinoma (Table 2). Median size of the malignant lesions was 14 mm (6–70). There was one patient who had a tumour-positive FNAC that could not be confirmed with histopathological analysis hereafter. At 3.8 years follow-up, no evidence of breast malignancy was found, and the lesion was therefore considered benign. Median follow-up of all patients without histopathology was 5.4 years (IQR 3.9–7.1).

**Table 2:** Pathological and radiological classification of malignant breast lesions

	Conventional imaging				MBI							
	BI-RADS ≤ 3		BI-RADS > 3		BI-RADS ≤ 3		BI-RADS > 3		Total		Size (mm)	
Ductal carcinoma	9	36%	6	24%	1	4%	14	56%	15	60%	15	6–70
Lobular carcinoma	3	12%	1	4%	2	8%	2	8%	4	16%	10	6–14
DCIS												
Grade 1	1	4%	–		–		1	4%	1	4%	9	–
Grade 2	2	8%	–		–		2	8%	2	8%	25	24–26
Grade 3	2	8%	–		1	4%	1	4%	2	8%	10	10–10
Intracystic carcinoma	0	–	1	4%	0		1	4%	1	4%	10	–
Total	17	68%	8	32%	4	16%	21	84%	25	100%	14	6–70

Data are median (range) or *N* (%). Conventional imaging includes mammography plus ultrasound.

DCIS, ductal carcinoma *in situ*; MBI, molecular breast imaging.

### Diagnostic accuracy of conventional imaging and molecular breast imaging

Overall, conventional diagnostics correctly assessed 8 [32%; 95% confidence interval (CI), 15–53] of 25 patients with malignant lesions as BI-RADS > 3, whereas MBI detected malignant lesions in 21 (84%; 95% CI, 64–95) of 25 patients (Table 2). MBI detected four of five patients with DCIS and eight of nine patients with a malignant lesion ≤1 cm, while none of these lesions were detected with conventional imaging (Table 2).

In patients with benign breast lesions, conventional imaging was correctly negative in 162 (81%; 95% CI, 74–86) of 201 patients and MBI was correctly negative in 173 (86%; 95% CI, 81–91) of 201 patients. Sensitivity of adjunct MBI was higher compared to conventional imaging (84% vs. 32%; *P* < 0.002), but specificity did not significantly differ (86% vs. 81%; *P* = 0.161). The positive predictive value (PPV) and negative predictive value (NPV) of MBI were 43% (95% CI, 29–58) and 98% (95% CI, 94–99), respectively, compared to 17% (95% CI, 8–31) and 91% (95% CI, 85–94) for conventional diagnostics (Table 3).

**Table 3:** Diagnostic accuracy of conventional imaging and molecular breast imaging in patients with indeterminate breast abnormalities and unresolved diagnostic concerns

	Malignant breast lesion		
	No (*n* = 201)	Yes (*n* = 25)	Total (*n* = 226)
Conventional imaging			
BI-RADS ≤ 3	162 (81%)	17 (68%)	179 (79%)
BI-RADS > 3	39 (19%)	8 (32%)	47 (21%)
MBI			
BI-RADS ≤ 3	173 (86%)	4 (16%)	177 (78%)
BI-RADS > 3	28 (14%)	21 (84%)	49 (22%)

Data are *n* (%). Conventional imaging mammography plus ultrasound.

MBI, molecular breast imaging.

### Clinical impact of molecular breast imaging

**Fig. 1:**
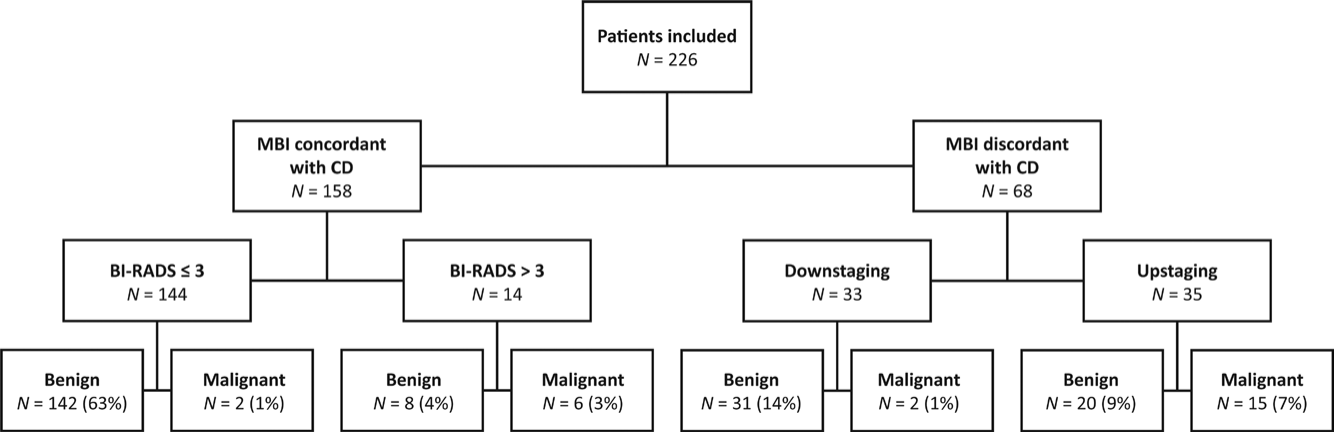
Value of adjunct molecular breast imaging in patients with remaining diagnostic concerns after conventional breast imaging. MBI, molecular breast imaging; CD, conventional diagnostic imaging with mammography and ultrasound. BI-RADS ≤ 3 classifications were considered benign and BI-RADS > 3 classifications were considered malignant. BI-RADS scores were compared with histopathological analysis or at least 6 months follow-up imaging.

**Fig. 2:**
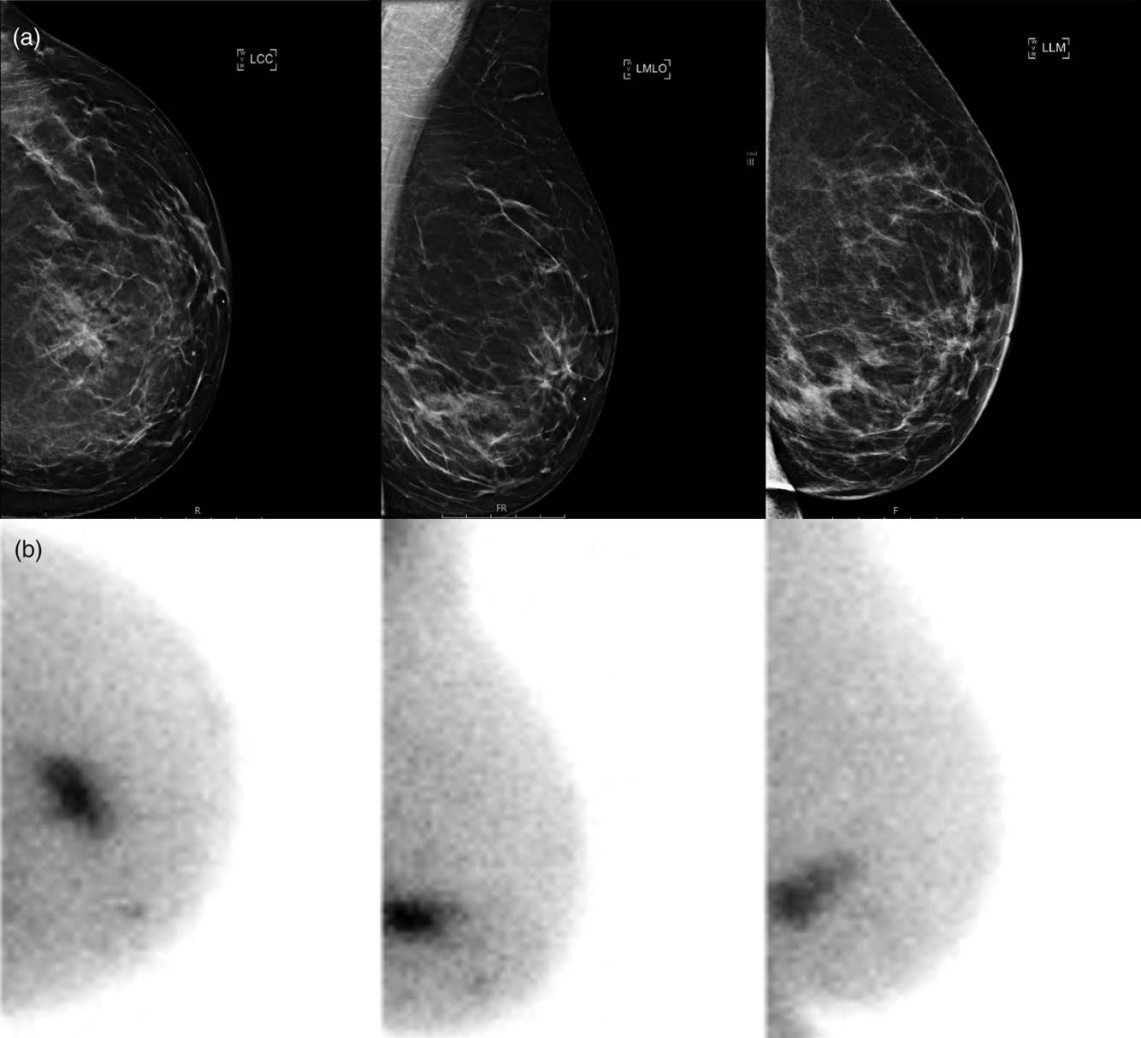
Breast imaging of a 56-year-old patient with left-sided breast cancer, detected at 1-year follow-up after treatment for right-sided breast cancer. With digital mammography, more glandular tissue was described in the medial lower quadrant (BI-RADS 3) (a), but no mass could be distinguished and ultrasound was negative. Molecular breast imaging (MBI; Dilon 6800 gamma camera) revealed suspicious sestamibi uptake in the medial lower quadrant over an area of 36 mm (b). MBI-guided biopsy was conducted, showing invasive carcinoma grade 2 with a lobular growth pattern, hormone receptor-positive, HER2-negative.

**Fig. 3:**
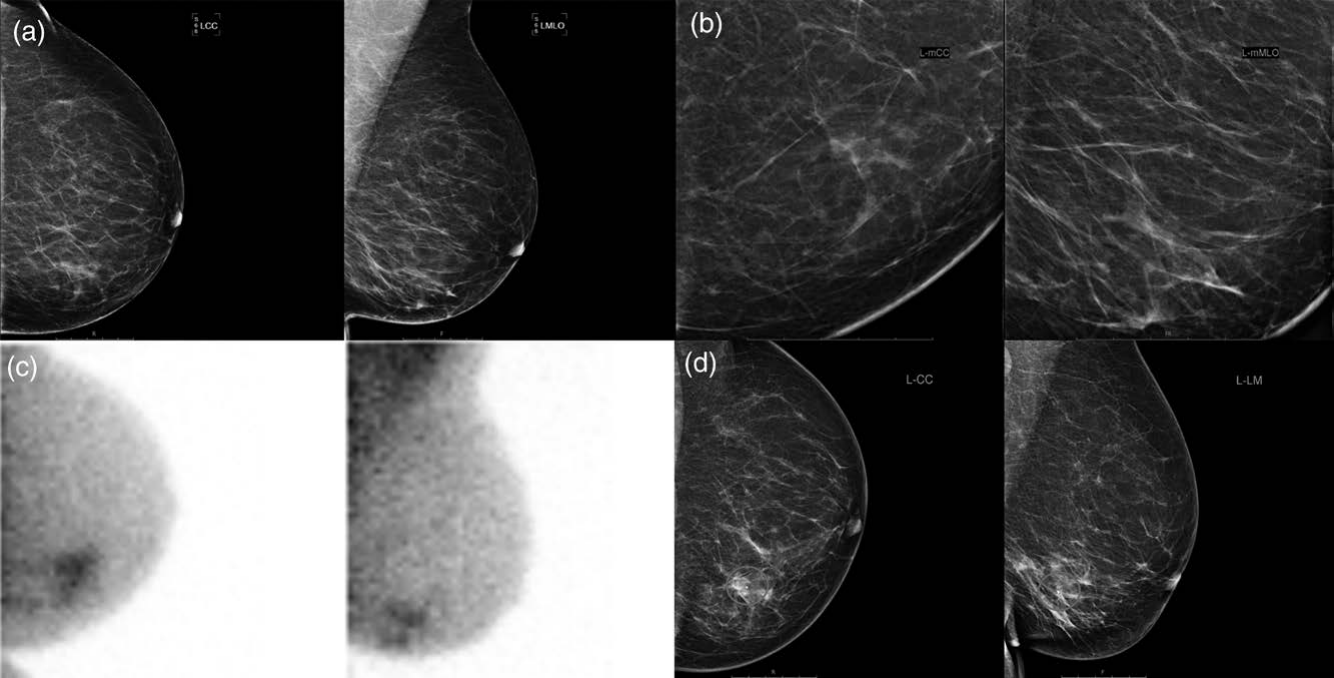
Breast imaging of a 69-year-old patient with left-sided breast cancer and ductal carcinoma *in situ*. The patient was referred by the national breast cancer screening program due to incomplete imaging (BI-RADS 0). There appeared to be slightly more glandular tissue in the medial lower quadrant on digital mammography (a) including the mammographic enlargements (b), with an inhomogeneous echotexture, but no mass could be distinguished. Adjunct molecular breast imaging showed suspicious irregular sestamibi uptake in the medial lower quadrant (c) (Dilon 6800 gamma camera). A hydromarker was placed in the area with inhomogeneous echotexture to confirm correlation with the pathological sestamibi uptake on MBI (d), after which ultrasound-guided biopsy was performed. The representativity of the acquired biopsy specimens was confirmed by showing uptake of sestamibi in the specimens in vitro. Pathological analysis showed ductal carcinoma in-situ grade 3 with a small focus of invasive carcinoma, hormone receptor-negative, HER2-positive. MBI, molecular breast imaging.

MBI categorised 68 of 226 (30%) patients in a different BI-RADS category than CONVENTIONAL imaging (i.e. BI-RADS ≤ 3: ‘benign’ or BI-RADS > 3: ‘malignant’) (Fig. 1). MBI downstaged 33 patients, of whom 27 patients underwent pathology analysis and 16 patients had follow-up imaging only. Two of 33 downstaged patients were diagnosed with malignant breast lesions and 31 (94%) patients had benign lesions. MBI upstaged 35 patients, of which 15 (43%) patients had malignant breast lesions; examples are shown in Figs 2 and 3. In 4 of 35 upstaged patients, pathology was not obtained as the MBI pattern was interpreted as corresponding with the clinical diagnosis of mastopathy.

In 14 of 16 of the remaining upstaged patients, histopathological findings were benign, meaning MBI-findings were false-positive in these patients. In three patients, histopathology was consistent with a fibroadenoma, five patients had changes consistent with mastopathy, and in the other six patients only normal breast tissue was found in the biopsy specimens. In two patients MBI was repeated after several weeks, since MBI-guided biopsy was technically impossible and lesions were occult on conventional diagnostic. Follow-up MBI showed significant decrease/absence of uptake, making benign aetiology more likely. Both patients remained in follow-up without evidence of malignancy during follow-up.

Overall, MBI correctly adjusted the imaging classification in 46 (68%; 95% CI, 55–78) of 68 patients in which MBI differed from conventional diagnostics, and was therefore of added value in 20% (95% CI, 15–46) of all patients (Fig. 1). Two of 25 patients with a malignant breast lesion (DCIS and lobular carcinoma) which were categorised BI-RADS ≤ 3 by both MBI and conventional imaging were diagnosed at short-term follow-up imaging.

### Characteristics associated with malignancy

Predictive for malignancy in patients presenting with an equivocal breast lesion or diagnostic concerns were older age [odds ratio (OR) 1.06; 95% CI, 1.02–1.10], a palpable breast lesion (OR 3.10; 95% CI, 1.07–9.03) and nipple discharge (OR 3.65; 95% CI, 1.18–11.28) compared to no symptoms, and the clinical classification ‘probably malignant’ (OR 4.16; 95% CI, 1.32–13.11) as assessed by a breast surgeon. At multivariate analysis, older age (OR 1.06; 95% CI, 1.02–1.10), a palpable breast lesion (OR 4.86; 95% CI, 1.14–20.66) and nipple discharge (OR 4.44; 95% CI, 1.13–17.50) remained predictive for in-situ or invasive breast cancer (Table 4).

**Table 4:** Predictive factors for breast malignancy

	Univariate			Multivariate		
	OR	95% CI	*P* value	OR	95% CI	*P* value
Age (years)	1.06	1.02–1.10	0.002	1.06	1.02–1.10	0.003
History of breast cancer	0.60	0.17–10	0.422			
Positive family history	0.82	0.29–2.30	0.702			
Presenting symptoms						
None	Ref.					
Pain	0.00	NA.	0.999	0.00	NA.	0.999
Palpable mass	3.10	1.07–9.03	0.038	4.86	1.14–20.66	0.032
Skin abnormality	1.55	0.17–14.20	0.698	2.16	0.19–25.20	0.538
Nipple discharge	3.65	1.18–11.28	0.025	4.44	1.13–17.50	0.033
Clinical classification						
No abnormalities	Ref.					
Benign	1.27	0.44–3.70	0.657	0.68	0.17–2.63	0.572
Uncertain benign	0.95	0.25–3.64	0.935	0.39	0.08–1.95	0.250
Uncertain malignant	4.16	1.32–13.11	0.015	1.15	0.26–5.06	0.858
Breast density						
Mostly fatty	Ref.					
Some density	0.70	0.24–2.06	0.519			
Moderately dense	0.49	0.25–1.57	0.230			
Extremely dense	0.34	0.04–3.12	0.343			

Univariate and multivariate logistic regressions.

CI, confidence interval; NA, not applicable; OR, odds ratio; Ref, reference.

### Value of molecular breast imaging in patients with nipple discharge

In the subgroup of 42 patients with nipple discharge, three patients had a different main indication for MBI: (1) referral by the national screening program with a BI-RADS 3 lesion, (2) to evaluate an additional MRI-detected lesion, and (3) to evaluate a post-surgical lesion. Sixteen patients presented with bloody nipple discharge.

In total, 8 (19%) of 42 patients were diagnosed with breast malignancy. Of the 16 patients with bloody nipple discharge, 5 (31%) patients had a malignant lesion. Conventional imaging categorised 40 of 42 patients as BI-RADS ≤ 3 and 2 patients as BI-RADS > 3. None of the 8 malignant lesions were correctly assessed by mammography and ultrasound. MBI upstaged 11 of 40 BI-RADS ≤ 3 patients, in which 7 (54%) patients with malignant lesions were found, and correctly downstaged the two patients that scored BI-RADS > 3 with conventional imaging. Thus, MBI correctly adjusted imaging classification in 9 (21%; 95% CI, 10–37) of 42 patients with nipple discharge. One of the eight malignant lesions was assessed BI-RADS ≤ 3 by both conventional imaging and MBI and was detected at follow-up imaging. Eleven (24%) of 42 patients with nipple discharge and negative conventional imaging and MBI were referred for ductoscopy, at which none of the patients were diagnosed with in-situ or invasive breast cancer.

Diagnostic sensitivity of MBI in patients with nipple discharge was 88% (7 of 8; 95% CI, 47–99). Specificity was lower for MBI than conventional imaging, although this was not statistically significant [82% (28/34) vs. 94% (32/34), *P* = 0.125]. PPV and NPV for MBI were 64% (7 of 11; 95% CI, 31–89) and 99% (28 of 29; 95% CI, 82–99), and 0% and 80% (32 of 40; 95% CI, 64–91) for conventional diagnostics, respectively.

### Value of molecular breast imaging in patients with BI-RADS 3 lesions

Eight (7%) out of 113 patients with BI-RADS 3 lesions at conventional diagnostics had malignant lesions. MBI upstaged 20 (18%) patients, of which 7 were true-positive for malignancy. One of the eight patients with malignant lesions was assessed BI-RADS 2 by MBI and was detected at follow-up imaging. MBI correctly categorised 99 (88%) of 113 patients with BI-RADS 3 lesions on conventional imaging in the ‘benign’ or ‘malignant’ category and correctly adjusted imaging classification in 7 (6%; 95% CI, 2–12) of 113 patients. Diagnostic sensitivity and specificity of MBI in patients with BI-RADS 3 lesions were 88% (7 of 8; 95% CI, 47–99) and 88% (92 of 105; 95% CI, 80–93), and PPV and NPV were 35% (7 of 20; 95% CI, 15–59) and 99% (92 of 93; 95% CI, 94–99).

## Discussion

MBI as additional diagnostic modality in patients with remaining concerns after the conventional work-up (i.e. mammography and ultrasound) greatly improved the diagnostic accuracy of imaging in this complex patient population. MBI identified significantly more malignant breast lesions compared to conventional imaging alone (84% vs. 32%) and detected 15 of 25 patients with (pre)malignant breast lesions that were missed at conventional imaging. Specificity of MBI was also higher compared to conventional diagnostics, although not statistically significant (86% vs. 81%). Overall, the addition of MBI in patients with equivocal breast lesions or diagnostic concerns led to a correct change in management in 20% of all patients.

Predictors for malignancy in this study population were older age, a palpable mass and nipple discharge. Pathological nipple discharge and especially bloody nipple discharge is a known predictor of breast cancer [23,24]. Therefore, we performed a subgroup analysis including 42 patients with remaining concerns and nipple discharge, in which MBI revealed malignant lesions in 19% of cases, all occult on mammography and ultrasound.

Subgroup analysis was also performed in patients with BI-RADS 3 breast lesions. This group represents a large proportion of patients with remaining diagnostic concerns after conventional work-up. For these patients, the American College of Radiology generally recommends 6 months follow-up imaging or diagnostic biopsy, if the patient has been referred by the breast cancer screening program [20]. We found that MBI correctly upstaged 6% of 113 patients with BI-RADS 3 lesions and particularly had a high NPV of 99%. Therefore, additional MBI could potentially allow omission of diagnostic biopsy procedures or discharge patients with BI-RADS 3 lesions from further follow-up.

Several studies have reported on the diagnostic accuracy of MBI in patients with proven breast cancer, as supplemental screening modality in patients with dense breast tissue or in women with an increased risk of breast cancer [11,12,15,16,25–30]. The clinical value of supplemental MBI for the detection of breast cancer in patients with diagnostic concerns; however, is less clear. Patient characteristics and indications for MBI vary across studies and few aimed to investigate the diagnostic accuracy of MBI as a problem-solving modality [18,19,31–40]. Notably, the incidence of (in-situ) breast cancer in all except one of these studies was much higher (7%–90%) than the incidence of breast cancer reported in the present study (11%), making it difficult to compare the results [18,19,31–37,39,40]. In addition, only three of these studies explicitly analysed (a subgroup of) patients with suspicious lesions at clinical examination that were negative with conventional imaging or patients with indeterminate breast lesions [18,19,32].

One of the three studies that explicitly investigated the value of MBI in patients with intermediate breast lesions or diagnostic concerns, was the study by Siegal *et al*. Siegal et al. retrospectively reviewed 416 cases in which MBI was ordered in more than half of the patients for an indeterminate asymmetry or focal asymmetry [32]. Other common indications were evaluation of calcifications, palpable lumps with negative imaging, and evaluation of patients with a surgical scar. In this study population, sensitivity and specificity of MBI were 93% and 79%, which slightly differs from the 84% and 86% we found [32]. This is not remarkable, as the incidence of breast malignancy was slightly lower (7% vs. 11%), the proportion of patients with intermediate lesions was higher (55% vs. 22%) and we did not include patients with calcifications.

Weigert *et al*. analysed 1042 patients in whom MBI was performed for at least two of the following indications: equivocal or negative conventional imaging and an unresolved clinical concern, personal history of breast cancer or current cancer diagnosis, palpable masses negative on conventional imaging, dense breast tissue or high risk for breast cancer [18]. Although the incidence of breast cancer was higher compared to both our study and the study by Siegal *et al*. (24% vs. 11% and 7%), the sensitivity and specificity of MBI were both lower, with rates of 80% and 75%, respectively [18,32]. However, this study population was also considerably different compared to our population, as Weigert *et al*. included patients with proven breast cancer and high-risk patients scheduled for breast cancer screening.

In the study by Spanu *et al*., a subgroup of 41 patients with suspicious lesions on physical examination or on ultrasound or MRI that were negative at mammography (BI-RADS ≤ 3) were analysed within a total study population of 467 patients [19]. MBI correctly identified malignancy in 31 of 33 patients with BI-RADS ≤ 3 lesions (sensitivity 94%), including 26 patients with heterogeneously/high-dense breast [19]. Both the sensitivity of MBI as well as the incidence of malignancy (33 of 41) were much higher in this study compared to our findings. Nevertheless, Spanu *et al*. reported substantial added value of MBI as adjunct diagnostic modality, as did the studies by Siegal *et al*. and Weigert *et al* [18,19,32].

Currently, MRI is the most commonly used supplemental diagnostic imaging modality in the evaluation of breast lesions [41]. MBI; however, has several advantages over MRI. These advantages include lower costs, quicker interpretation and greater cost-effectiveness [27,42]. In addition, MBI can also be used for patients with obesity, metal implants or claustrophobia, in contrast to MRI. Maybe the most important advantage of MBI over MRI is its higher specificity, leading to a significantly higher PPV of additional biopsies [43]. Furthermore, MBI also provides the possibility of obtaining MBI-guided biopsies. In case of MBI-guided biopsy, the representativeness of the obtained biopsy specimen can be verified by measuring its radioactivity, a verification step that is not available in MRI-guided biopsy [22,44]. In the current study, in all 14 patients who underwent both MBI as well as MRI, MBI- and MRI-findings were concordant, supporting the non-inferiority of MBI to MRI.

The use of MBI has been criticised because it did not seem to be sensitive enough in detecting small, sub-centimetre breast lesions. New improvements in breast-dedicated gamma cameras; however, have resulted in improved images with a smaller dosage of radiotracer administered [13,35]. Several studies have now confirmed the accuracy of MBI in patients with small-breast tumours [17,31]. Our study confirms these findings, as 8 of 9 malignant breast lesions of less than 1 cm were correctly identified.

An often brought up limitation of MBI is the fact that it requires injection of a radiotracer, resulting in radiation exposure to the patient. In our centre, patients received 600 MBq (16 mCi) of radiotracer until 2018, resulting in a whole-body radiation dose of 5 mSv distributed throughout the body. Albeit this is similar to the effective dose of other commonly applied nuclear diagnostic examinations, such as cardiac imaging and bone scintigraphy, it is higher than the effective dose of digital mammography (0.5 mSv), although in this case the dose is concentrated to the breasts [40]. Recently, low-dose imaging protocols have been described [30], and technological innovations have resulted in the availability of more sensitive (CZT-dual head) MBI systems, allowing a significant reduction of the absorbed dose to the breast (0.25 mGy, 1.1 mSv) [45]. As a result, MBI may be opted as screening tool in patients with dense breast tissue [13,16]. In our institute, these developments have resulted in a reduction of the administered activity to 200 MBq (5 mCi).

Possible limitations of our study are its retrospective and single-centre character, the use of MBI in a population with divergent diagnostic dilemmas and the fact that not all patients underwent pathological assessment of the breast lesions. However, a major advantage of our study is its long-term follow-up. No other malignant breast lesions were detected at a median follow-up of more than 5 years, which practically nullifies the missing pathological assessments of patients categorised as benign with adjunct MBI.

In this study, we found that MBI was of great additional value in patients with remaining diagnostic concerns after the conventional work-up with mammography and ultrasound, due to its high NPV and good PPV of 98% and 43%, respectively. MBI could accurately rule out malignancy, especially in the subpopulation of patients with BIRADS 3 lesions, and identified a significant number of (pre)malignant lesions overlooked with conventional imaging alone. Therefore, we recommend the use of MBI as adjunct modality to guide decision-making (e.g. obtaining pathology or follow-up imaging) in patients with undetermined breast abnormalities and unresolved diagnostic concerns after conventional imaging.

## Acknowledgements

### Conflicts of interest

There are no conflicts of interest.

## References

[1] Veronesi P De Lorenzi F Loschi P Rietjens M Veronesi U. Erratum to: current trends in the oncologic and surgical managements of breast cancer in women with implants: incidence, diagnosis, and treatment. Aesthetic Plast Surg 2016; 40:810. 27389406 10.1007/s00266-016-0679-9

[2] Prummel MV Muradali D Shumak R Majpruz V Brown P Jiang H. Digital compared with screen-film mammography: measures of diagnostic accuracy among women screened in the ontario breast screening program. Radiology 2016; 278:365–373. 26334680 10.1148/radiol.2015150733

[3] Bakker MF de Lange SV Pijnappel RM Mann RM Peeters PHM Monninkhof EM.; DENSE Trial Study Group. Supplemental MRI screening for women with extremely dense breast tissue. N Engl J Med 2019; 381:2091–2102. 31774954 10.1056/NEJMoa1903986

[4] Greenwood HI Freimanis RI Carpentier BM Joe BN. Clinical breast magnetic resonance imaging: technique, indications, and future applications. Semin Ultrasound CT MR 2018; 39:45–59. 29317039 10.1053/j.sult.2017.07.002

[5] Knuttel FM Menezes GL van den Bosch MA Gilhuijs KG Peters NH. Current clinical indications for magnetic resonance imaging of the breast. J Surg Oncol 2014; 110:26–31. 24861355 10.1002/jso.23655

[6] Mann RM Cho N Moy L. Breast MRI: state of the art. Radiology 2019; 292:520–536. 31361209 10.1148/radiol.2019182947

[7] Houssami N Ciatto S Macaskill P Lord SJ Warren RM Dixon JM. Accuracy and surgical impact of magnetic resonance imaging in breast cancer staging: systematic review and meta-analysis in detection of multifocal and multicentric cancer. J Clin Oncol 2008; 26:3248–3258. 18474876 10.1200/JCO.2007.15.2108

[8] Onega T Weiss JE Goodrich ME Zhu W DeMartini WB Kerlikowske K. Relationship between preoperative breast MRI and surgical treatment of non-metastatic breast cancer. J Surg Oncol 2017; 116:1008–1015. 29127715 10.1002/jso.24796 PMC5760434

[9] Zhang M Sun S Mesurolle B. The impact of pre-operative breast MRI on surgical waiting time. PLoS One 2017; 12:e0169756. 28068382 10.1371/journal.pone.0169756 PMC5221790

[10] Huppe AI Mehta AK Brem RF. Molecular breast imaging: a comprehensive review. Semin Ultrasound CT MR 2018; 39:60–69. 29317040 10.1053/j.sult.2017.10.001

[11] Yu X Hu G Zhang Z Qiu F Shao X Wang X. Retrospective and comparative analysis of (99m)Tc-Sestamibi breast specific gamma imaging versus mammography, ultrasound, and magnetic resonance imaging for the detection of breast cancer in Chinese women. BMC Cancer 2016; 16:450. 27401536 10.1186/s12885-016-2537-1 PMC4940883

[12] Choi EK Im JJ Park CS Chung YA Kim K Oh JK. Usefulness of feature analysis of breast-specific gamma imaging for predicting malignancy. Eur Radiol 2018; 28:5195–5202. 29948076 10.1007/s00330-018-5563-3

[13] Rhodes DJ Hruska CB Conners AL Tortorelli CL Maxwell RW Jones KN. Journal club: molecular breast imaging at reduced radiation dose for supplemental screening in mammographically dense breasts. AJR Am J Roentgenol 2015; 204:241–251. 25615744 10.2214/AJR.14.13357 PMC4423604

[14] Buscombe JR Cwikła JB Thakrar DS Hilson AJ. Scintimammography: a review. Nucl Med Rev Cent East Eur 1999; 2:36–41. 14600999

[15] Holbrook A Newel MS. Alternative screening for women with dense breasts: breast-specific gamma imaging (molecular breast imaging). AJR Am J Roentgenol 2015; 204:252–256. 25615745 10.2214/AJR.14.13525

[16] Kim BS Moon BI Cha ES. A comparative study of breast-specific gamma imaging with the conventional imaging modality in breast cancer patients with dense breasts. Ann Nucl Med 2012; 26:823–829. 22922890 10.1007/s12149-012-0649-5

[17] Sun Y Wei W Yang HW Liu JL. Clinical usefulness of breast-specific gamma imaging as an adjunct modality to mammography for diagnosis of breast cancer: a systemic review and meta-analysis. Eur J Nucl Med Mol Imaging 2013; 40:450–463. 23151912 10.1007/s00259-012-2279-5

[18] Weigert JM Bertrand ML Lanzkowsky L Stern LH Kieper DA. Results of a multicenter patient registry to determine the clinical impact of breast-specific gamma imaging, a molecular breast imaging technique. AJR Am J Roentgenol 2012; 198:W69–W75. 22194518 10.2214/AJR.10.6105

[19] Spanu A Sanna D Chessa F Manca A Cottu P Fancellu A. The clinical impact of breast scintigraphy acquired with a breast specific gamma-camera (BSGC) in the diagnosis of breast cancer: incremental value versus mammography. Int J Oncol 2012; 41:483–489. 22641247 10.3892/ijo.2012.1495

[20] D’Orsi CJ Sickles EA Mendelson EB Morris EA. ACR BI-RADS® Atlas, breast imaging reporting and data system. Reston, VA: American College of Radiology; 2013.

[21] Conners AL Hruska CB Tortorelli CL Maxwell RW Rhodes DJ Boughey JC. Lexicon for standardized interpretation of gamma camera molecular breast imaging: observer agreement and diagnostic accuracy. Eur J Nucl Med Mol Imaging 2012; 39:971–982. 22289959 10.1007/s00259-011-2054-z

[22] Collarino A Valdes Olmos RA van der Hoeven AF Pereira Arias-Bouda LM. Methodological aspects of (99m)Tc-sestamibi guided biopsy in breast cancer. Clin Transl Imaging 2016; 4:367–376. 27738627 10.1007/s40336-016-0201-z PMC5037160

[23] Chen L Zhou WB Zhao Y Liu XA Ding Q Zha XM. Bloody nipple discharge is a predictor of breast cancer risk: a meta-analysis. Breast Cancer Res Treat 2012; 132:9–14. 21947751 10.1007/s10549-011-1787-5

[24] Zacharioudakis K Kontoulis T Vella JX Zhao J Ramakrishnan R Cunningham DA. Can we see what is invisible? The role of MRI in the evaluation and management of patients with pathological nipple discharge. Breast Cancer Res Treat 2019; 178:115–120. 31352554 10.1007/s10549-019-05321-w PMC6790184

[25] Tan H Zhang H Yang W Fu Y Gu Y Du M. Breast-specific gamma imaging with Tc-99m-sestamibi in the diagnosis of breast cancer and its semiquantitative index correlation with tumor biologic markers, subtypes, and clinicopathologic characteristics. Nucl Med Commun 2016; 37:792–799. 27058361 10.1097/MNM.0000000000000518

[26] Shermis RB Wilson KD Doyle MT Martin TS Merryman D Kudrolli H. Supplemental breast cancer screening with molecular breast imaging for women with dense breast tissue. AJR Am J Roentgenol 2016; 207:450–457. 27186635 10.2214/AJR.15.15924

[27] Hruska CB Conners AL Jones KN O’Connor MK Moriarty JP Boughey JC. Diagnostic workup and costs of a single supplemental molecular breast imaging screen of mammographically dense breasts. AJR Am J Roentgenol 2015; 204:1345–1353. 26001247 10.2214/AJR.14.13306 PMC5036572

[28] Hruska CB. Molecular breast imaging for screening in dense breasts: state of the art and future directions. AJR Am J Roentgenol 2017; 208:275–283. 27762607 10.2214/AJR.16.17131

[29] Hendrick RE Tredennick T. Benefit to radiation risk of breast-specific gamma imaging compared with mammography in screening asymptomatic women with dense breasts. Radiology 2016; 281:583–588. 27257949 10.1148/radiol.2016151581

[30] Brem RF Ruda RC Yang JL Coffey CM Rapelyea JA. Breast-specific gamma-imaging for the detection of mammographically occult breast cancer in women at increased risk. J Nucl Med 2016; 57:678–684. 26823569 10.2967/jnumed.115.168385

[31] O’Connor MK Phillips SW Hruska CB Rhodes DJ Collins DA. Molecular breast imaging: advantages and limitations of a scintimammographic technique in patients with small breast tumors. Breast J 2007; 13:3–11. 17214787 10.1111/j.1524-4741.2006.00356.x

[32] Siegal E Angelakis E Morris P Pinkus E. Breast molecular imaging: a retrospective review of one institutions experience with this modality and analysis of its potential role in breast imaging decision making. Breast J 2012; 18:111–117. 22300043 10.1111/j.1524-4741.2011.01214.x

[33] Brem RF Floerke AC Rapelyea JA Teal C Kelly T Mathur V. Breast-specific gamma imaging as an adjunct imaging modality for the diagnosis of breast cancer. Radiology 2008; 247:651–657. 18487533 10.1148/radiol.2473061678

[34] Lee A Chang J Lim W Kim BS Lee JE Cha ES. Effectiveness of breast-specific gamma imaging (BSGI) for breast cancer in Korea: a comparative study. Breast J 2012; 18:453–458. 22897514 10.1111/j.1524-4741.2012.01280.x

[35] Kuhn KJ Rapelyea JA Torrente J Teal CB Brem RF. Comparative diagnostic utility of low-dose breast-specific gamma imaging to current clinical standard. Breast J 2016; 22:180–188. 26662297 10.1111/tbj.12550

[36] Meissnitzer T Seymer A Keinrath P Holzmannhofer J Pirich C Hergan K. Added value of semi-quantitative breast-specific gamma imaging in the work-up of suspicious breast lesions compared to mammography, ultrasound and 3-T MRI. Br J Radiol 2015; 88:20150147. 25882690 10.1259/bjr.20150147 PMC4628538

[37] Schillaci O Cossu E Romano P Sanso C Danieli R Granai AV. High-resolution gamma-camera for molecular breast imaging: First clinical results. Phys Med 2006; 21(Suppl 1):121–124. 17646012 10.1016/S1120-1797(06)80042-6

[38] Shermis RB Redfern RE Burns J Kudrolli H. Molecular Breast imaging in breast cancer screening and problem solving. Radiographics 2017; 37:1309–1606. 28898193 10.1148/rg.2017160204

[39] Chung HW So Y Yang JH Park KS Yoo YB Choi N. Adjunctive breast-specific gamma imaging for detecting cancer in women with calcifications at mammography. Ann Surg Oncol 2017; 24:3541–3548. 28819909 10.1245/s10434-017-6058-1

[40] Cho MJ Yang JH Yu YB Park KS Chung HW So Y. Validity of breast-specific gamma imaging for Breast Imaging Reporting and Data System 4 lesions on mammography and/or ultrasound. Ann Surg Treat Res 2016; 90:194–200. 27073789 10.4174/astr.2016.90.4.194 PMC4826981

[41] Mann RM Kuhl CK Kinkel K Boetes C. Breast MRI: guidelines from the European Society of Breast Imaging. Eur Radiol 2008; 18:1307–1318. 18389253 10.1007/s00330-008-0863-7 PMC2441490

[42] Johnson N Sorenson L Bennetts L Winter K Bryn S Johnson W. Breast-specific gamma imaging is a cost effective and efficacious imaging modality when compared with MRI. Am J Surg 2014; 207:698–701; discussion 701. 24791629 10.1016/j.amjsurg.2013.12.015

[43] Sumkin JH Berg WA Carter GJ Bandos AI Chough DM Ganott MA. Diagnostic performance of MRI, molecular breast imaging, and contrast-enhanced mammography in women with newly diagnosed breast cancer. Radiology 2019; 293:531–540. 31660801 10.1148/radiol.2019190887

[44] Collarino A Olmos RAV Neijenhuis PA den Hartog WC Smit F de Geus-Oei LF. First clinical experience using stereotactic breast biopsy guided by (99m)Tc-Sestamibi. AJR Am J Roentgenol 2017; 209:1367–1373. 28379735 10.2214/AJR.17.18083

[45] O’Connor MK. Molecular breast imaging: an emerging modality for breast cancer screening. Breast Cancer Manag 2015; 4:33–40. 25621015 10.2217/BMT.14.49 PMC4303579

